# The Simple prEservatioN of Single cElls method for cryopreservation enables the generation of single-cell immune profiles from whole blood

**DOI:** 10.3389/fimmu.2023.1271800

**Published:** 2023-11-28

**Authors:** Sarthak Satpathy, Beena E. Thomas, William J. Pilcher, Mojtaba Bakhtiari, Lori A. Ponder, Rafal Pacholczyk, Sampath Prahalad, Swati S. Bhasin, David H. Munn, Manoj K. Bhasin

**Affiliations:** ^1^ Aflac Cancer and Blood Disorders Center, Children Healthcare of Atlanta, Atlanta, GA, United States; ^2^ Department of Biomedical Informatics, Emory University, Atlanta, GA, United States; ^3^ Department of Pediatrics, Emory University, Atlanta, GA, United States; ^4^ Division of Rheumatology, Children’s Healthcare of Atlanta, Atlanta, GA, United States; ^5^ Georgia Cancer Center, Augusta University, Augusta, GA, United States; ^6^ Department of Biochemistry and Molecular Biology, Augusta University, Augusta, GA, United States; ^7^ Department of Human Genetics, Emory University School of Medicine, Atlanta, GA, United States; ^8^ Department of Pediatrics, Augusta University, Augusta, GA, United States

**Keywords:** whole blood, cryopreservation, single cell profiling, density gradient, scRNA seq

## Abstract

**Introduction:**

Current multistep methods utilized for preparing and cryopreserving single-cell suspensions from blood samples for single-cell RNA sequencing (scRNA-seq) are time-consuming, requiring trained personnel and special equipment, so limiting their clinical adoption. We developed a method, Simple prEservatioN of Single cElls (SENSE), for single-step cryopreservation of whole blood (WB) along with granulocyte depletion during single-cell assay, to generate high quality single-cell profiles (SCP).

**Methods:**

WB was cryopreserved using the SENSE method and peripheral blood mononuclear cells (PBMCs) were isolated and cryopreserved using the traditional density-gradient method (PBMC method) from the same blood sample (n=6). The SCPs obtained from both methods were processed using a similar pipeline and quality control parameters. Further, entropy calculation, differential gene expression, and cellular communication analysis were performed to compare cell types and subtypes from both methods.

**Results:**

Highly viable (86.3 ± 1.51%) single-cell suspensions (22,353 cells) were obtained from the six WB samples cryopreserved using the SENSE method. In-depth characterization of the scRNA-seq datasets from the samples processed with the SENSE method yielded high-quality profiles of lymphoid and myeloid cell types which were in concordance with the profiles obtained with classical multistep PBMC method processed samples. Additionally, the SENSE method cryopreserved samples exhibited significantly higher T-cell enrichment, enabling deeper characterization of T-cell subtypes. Overall, the SENSE and PBMC methods processed samples exhibited transcriptional, and cellular communication network level similarities across cell types with no batch effect except in myeloid lineage cells.

**Discussion:**

Comparative analysis of scRNA-seq datasets obtained with the two cryopreservation methods i.e., SENSE and PBMC methods, yielded similar cellular and molecular profiles, confirming the suitability of the former method’s incorporation in clinics/labs for cryopreserving and obtaining high-quality single-cells for conducting critical translational research.

## 1 Introduction

Recent advances in single-cell microfluidic technologies have resulted in a ubiquitous implementation of single-cell approaches to understand disease mechanisms and developmental biology (1–3). Single-cell assays provide high-resolution measurement of cell types/subtypes (4) and their molecular states associated with health/disease conditions (5). Single-cell assays have immense potential in the discovery of cell-specific biomarkers (6) and for gaining unprecedented insights into composite cell-to-cell interactions that drive therapeutic responses (7) for expanding disease diagnosis and therapeutic options (8). We are utilizing single cell assays for the development of single-cell atlases for multiple myeloma (MM) (9, 10), pediatric cancers (11) as well as chronic wounds (12–14), to identify next-generation prognostic biomarkers with high sensitivity and specificity. Recently, a comparative analysis by our group, of rapid and non-progressing MM patient samples using single-cell profiling (SCP), revealed a significant contribution of exhausted T-cells in the rapid progression of MM (9). The implementation of SCP in another study on diabetic foot ulcers (DFUs) resulted in the identification of a unique fibroblast population associated with the healing of chronic DFUs in diabetic patients (14). A major issue with the single-cell approach is that samples need to be immediately subjected to downstream processing for live cell capture or frozen viably, both of which require precious time and bench-work, often not feasible in a clinical setting. Therefore, developing and optimizing methodologies that enable stable cryopreservation of clinic/hospital-collected samples with minimal intervention is crucial for implementation of SCP assays as routine.

**Figure 1:**
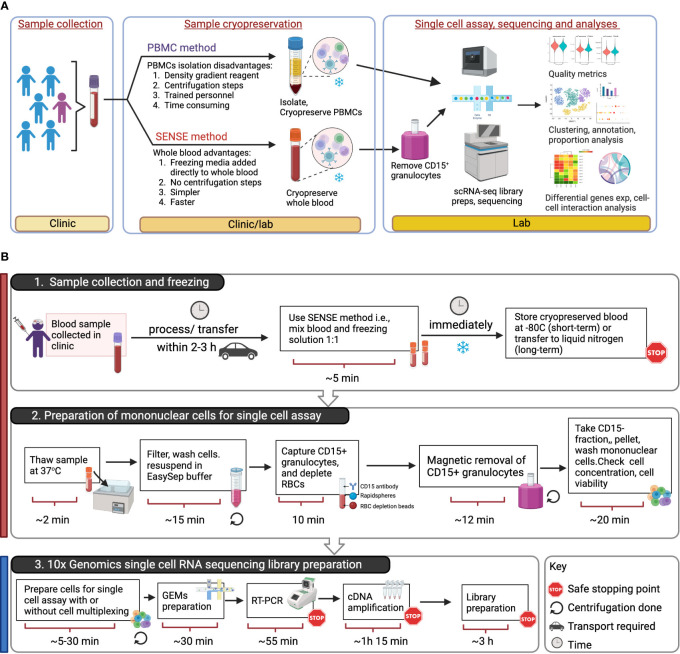
Overview of SENSE (Simple PrEservatioN of Single cElls) method for cryopreservation and single-cell immune profiles from whole blood. **(A)** Assay Overview: Blood samples were collected in EDTA tubes which were then split into two aliquots. One aliquot was processed using the traditional Ficoll-Paque density gradient method to isolate PBMCs, which were then cryopreserved. The other aliquot of blood was viably cryopreserved using the SENSE method, i.e., a cryoprotectant solution was added and the sample frozen. Post-thawing, the WB cells from the SENSE method were subjected to granulocyte depletion, and the CD15^-^ fraction was collected, washed, and processed for single-cell profiling using the 10x Genomics method. The resulting single-cell data from the SENSE and PBMC methods were then compared to identify any differences in cell quality metrics and molecular profiles. **(B)** Schematic for single cell assays using sample stored and processed using the SENSE method involving: 1. simple cryopreservation of whole blood samples stably stored for short or long term in -80 °C or liquid nitrogen respectively, and 2. preparation of sample for single cell assay by removing granulocytes and RBCs. 3. Generation of single cell RNA sequencing libraries using appropriate 10x Genomics kits (5’ or 3’with/without cell multiplexing). The color bars on the left-hand side serve as visual indicators, with the red bar denoting steps specific to SENSE method, while the blue bar represents steps common single cell profiling steps for both methods. The figure was prepared using BioRender.com.

Sample preparation for bulk sequencing can be performed on samples collected in the clinic in tubes with RNA/DNA stabilizers (15) without the need for immediate pre-processing (16). Although this approach is easy and practical in a clinical setting, a major drawback is that bulk approaches only reveal the average behavior of all the different cell populations in a sample (17). On the contrary, single-cell assays measure individual cell profiles (18) and their transcriptional states in the complex tissue architecture (2). However, the inherent need for viable cells (19) for performing single-cell assays limits the adoption of single-cell assays in clinics (bench to bedside) as well as the collection of clinical samples for single-cell research (bedside to bench). The traditional method of isolating PBMCs involves multiple centrifugation steps, the addition of special density gradient reagents like Ficoll-Paque to facilitate the isolation of mononuclear cells (20) for downstream single-cell assays and significant time commitment (**Figure 1A**). These preprocessing steps to isolate PBMCs may inevitably delay sample cryopreservation, which can potentially introduce technical bias and artifacts during SCP (21). To overcome the limitations associated with traditional sample preservation for single-cell assays, we have developed and implemented the **S**imple pr**E**servatio**N** of **S**ingle c**E**lls **(SENSE**) method for one-step cryopreservation of whole blood (WB) by the direct addition of freezing solution. The SENSE method also incorporates a granulocyte removal step during single-cell assay steps, resulting in optimal capture of immune repertoire from WB samples. In this study for the first time, we have performed a deep characterization of the SENSE method-generated transcriptome profiles and compared it with the transcriptome profiles of the PBMCs isolated by the standard Ficoll-Paque gradient method. Comparative analysis was performed on the patient blood samples collected in clinic setting to pave the way for the clinical implementation of the SENSE method. Development and implementation of simplified cryopreservation of WB samples using methods like one-step SENSE method would result in a significant increase in the adoption of SCP in clinics and single/multi-center therapeutic trials and enable robust identification of next-generation diagnostics, prognostics, and therapeutic biomarkers.

## 2 Results

### 2.1 Whole blood cryopreservation by the SENSE method generated high-quality cells

We tested the feasibility and performance of the SENSE method for SCP on whole blood samples, collected in a clinic at Children’s Healthcare of Atlanta from Juvenile Idiopathic Arthritis (JIA) (n=5) and pediatric lupus (n=1) patients (**Table 1**). The blood samples (3.0 ml - 4.5 ml) were split into two equal aliquots and processed in parallel using the SENSE and PBMC methods. The Ficoll-Paque density gradient method was used to isolate PBMCs from one-half of the sample which were then frozen in freezing media (Fisher Scientific). These viably frozen PBMCs were thawed and profiled directly using 10x Genomics Next GEM single cell 3’v3.1 kits (as described in the methods section) (**Figure 1A**). The remaining half of the blood sample was processed using the SENSE method that involved freezing WB directly by adding freezing solution (80% FBS, 20% DMSO) at a ratio of 1:1 to obtain a final concentration of 40% FBS, 10% DMSO in the cryopreserved WB samples. In the SENSE method, frozen WB was thawed, and the mononuclear cells were collected and resuspended after the removal of CD15^+^ and red blood cells as described in the detailed protocol (**Supplementary Document 1**; **Figure 1B**). The cells were then used for generating single-cell RNA sequencing (scRNA-seq) libraries. The data obtained from both methods were extensively studied by comparing various qualitative and quantitative parameters (**Figure 1A**). Simple single-step cryopreservation of WB made possible with incorporation of the SENSE method will promote the clinical implementation of SCP assays and expand single-cell research and discoveries (**Supplementary Figure 1**).

**Table 1:** Patient characteristics table.

Patient no.	Diagnosis age (years)	Sample collection age (years)	Sex	Race	Ethnicity	Diagnosis
1	6	7	Male	White	Non-hispanic	Oligoarticular JIA
2	2	16	Female	White	Non-hispanic	Polyarticular RF-
3	8	13	Female	White	Non-hispanic	Unfifferntiated JIA
4	5	11	Male	AA/black	Non-hispanic	Systemic JIA
5	1	8	Female	Asian	Non-hispanic	Polyarticular RF+
6	15	16	Female	AA/black	Non-hispanic	Lupus

JIA, Juvenile idiopathic arthritis; RF, Rheumatoid factor. Patient 1-5: JIA, Patient 6: Pediatric lupus.

**Figure 2:**
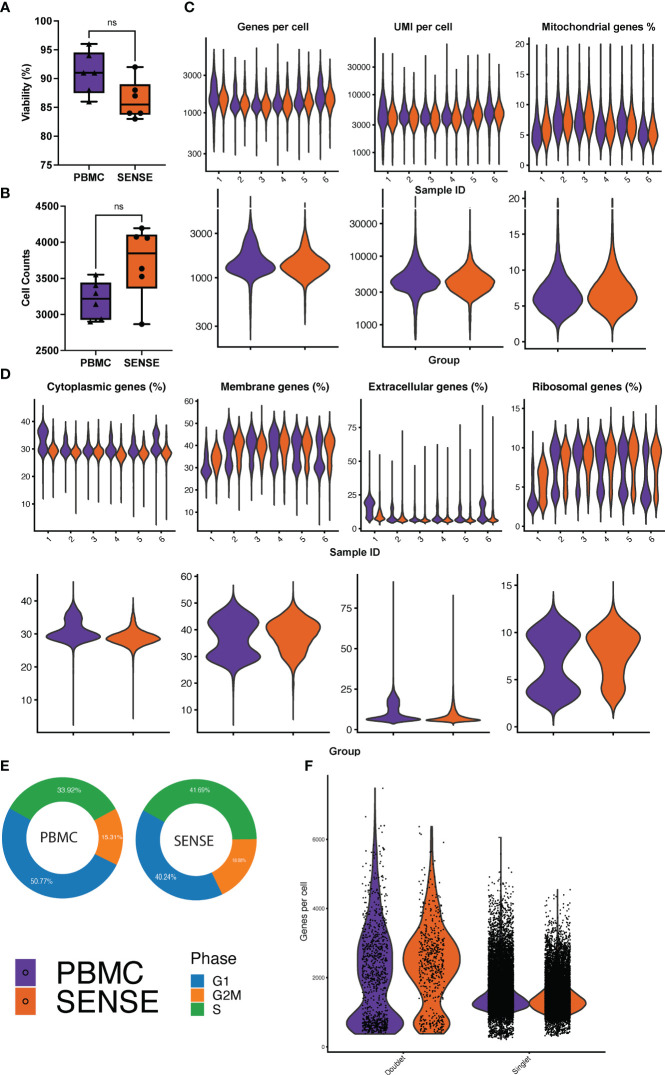
Comparative analysis of cell quality of SENSE and PBMCs methods. **(A)** Cell viability % boxplots, and **(B)** Single-cell counts boxplots with each dot representing an individual patient. The significance of the difference between the methods was tested using the paired Student’s t-test. NS indicates non-significant differences with *P* >.05. **(C)** Count of Genes (log-scale), UMIs (log-scale), and proportion of mitochondrial genes per cell. The violin plots in the top panel show patient-wise information for the count of genes, UMIs, and proportion of mitochondrial genes per cell, while the violin plots in the bottom panel show the group-wise comparison of SENSE and PBMC methods. **(D)** Proportion of patient and group-wise genes in cytoplasmic, membrane, extracellular, and ribosomal gene ontology categories. **(E)** Proportion of cells from SENSE and PBMC methods in the G1, G2M, and S phases, and **(F)** Proportion of the singlets and doublets cells in the SENSE and PBMCs protocols.

Comparative analysis of cell viability upon thawing of cryopreserved WB followed by granulocyte depletion (SENSE method) and thawing of cryopreserved PBMCs (density gradient method) revealed that the latter method yielded slightly higher viability (91 ± 1.64%) as compared to the former method (86.3 ± 1.51%), however, this difference was not significant (*P*=.10) (**Figure 2A**). A total of 20,024 and 23,502 cells were profiled from the PBMCs isolated using density gradient method and WB frozen using SENSE method respectively, hereby referred to as ‘PBMC’ and ‘SENSE’ for simplicity. The low-quality cell identification based on unique genes (<200), UMI count (<600), and mitochondrial transcripts (>20%) identified 793 and 1,149 low-quality cells with PBMC and SENSE methods respectively, that were filtered out from the subsequent analysis. This resulted in 19,231 and 22,353 high-quality cells from the PBMC and SENSE methods respectively (**Figure 2B**). The SENSE method was found to capture median gene counts and unique molecular identifiers (UMIs) comparable to the PBMC method, with a similar median representation of mitochondrial genes (**Figure 2C**). To check whether the SENSE method affected the integrity of the cells, the representation of genes in the membrane, extracellular, and ribosomal ontology categories were assessed. Cellular damage results in increasing the representation of the membrane genes and lowering the representation of extracellular genes (22). Comparative analysis showed profiling of similar proportions of cytoplasmic, membrane, extracellular, and ribosomal ontology categories with SENSE and PBMC methods, demonstrating that the former method is as robust as the latter method in obtaining high-quality cells from WB with no introduction of cellular damage artifacts (**Figure 2D**). SCP can be utilized to evaluate the cell cycle phases (i.e., G1, G2/M, and S) which significantly impact cellular gene expression and are vital in classifying cellular sub-populations in the single-cell assays. The comparative analysis revealed a broadly similar distribution of cells in various cell cycle phases between cells from all samples from the SENSE and PBMC methods (**Figure 2E**). Additionally, we also assessed the impact of sample processing by each method on the doublet rates as they are key confounders in the single cell data (23). The WB samples cryopreserved using the SENSE method had a lower percentage of doublets (2.41%, 539 cells) as compared to the PBMC method (4.76%, 916 cells) (**Figure 2F**), demonstrating that single cells cryopreserved using the SENSE method generated high-quality SCPs of clinical WB samples. Clusters with a high percentage of doublets were manually reviewed using canonical marker expression and excluded from downstream analysis.

Altogether, these single-cell quality assessment analyses demonstrate that the SENSE method is a reliable and effective method for WB single-cell profiling by preserving high-quality cells that yield comparable results to the traditional density gradient PBMCs isolation method.

### 2.2 Cellular profile and enrichment between SENSE and PBMCs methods

**Figure 3:**
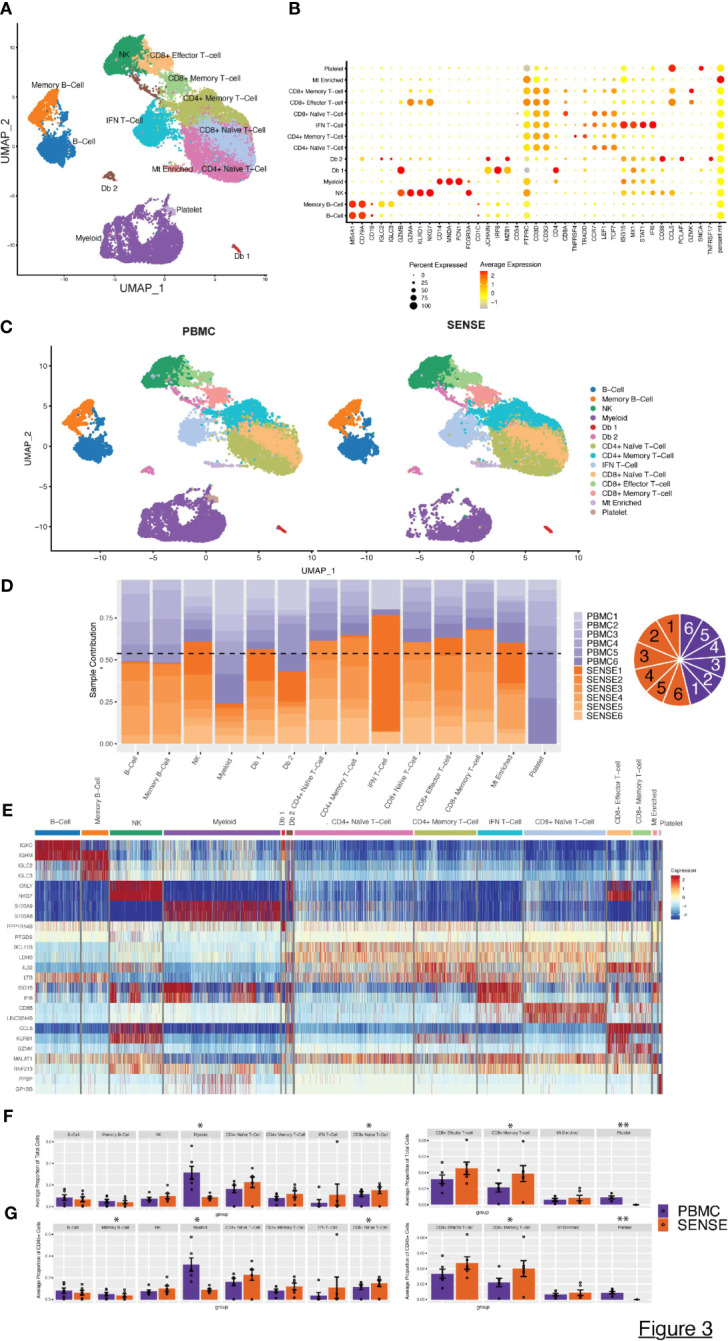
Characterization of blood single cell profiles obtained with SENSE and PBMCs methods. Blood samples for the analysis were obtained from JIA and pediatric Lupus patients from the rheumatology clinic in CHOA. **(A)** Uniform Manifold Approximation and Projection (UMAP) embedding of scRNA-seq data from both methods across all patients consisting of >41,000 high-quality single-cells distributed into 11 cell types. Canonical cell types are based on the expression of marker genes that include: B-Cells (*MS4A1* ^+^, *CD79A* ^+^), Memory B-cells (*CD19^+^, IGLC2* ^+^), NK Cells (*NKG7* ^+^, *KLRD1* ^+^, *CD3D* ^-^), Myeloid cells (CD14^+^, MNDA^+^, FCGR3A^+^, FCN^+^), CD4+ Naïve T-cells (*CD3D* ^+^, *CD4^+^, CCR7^+^, LEF1^+^*), CD4^+^ Memory T-cells (*CD3D* ^+^, *CD4^+^, TRAAD^+^, TNFRSF4^+^*), IFN T cells (*CD3D* ^+^, ISG15*^+^*, STAT1*^+^*, IFI6*^+^*), CD8^+^ Effector T-cells (*GZMA* ^+^, *GZMB* ^+^, *CD8A* ^+^), CD8^+^ Naive T-cells (*CD8A* ^+^, *CCR7* ^+^, *LEF1* ^+^, *TCF7* ^+^), CD8^+^ Memory T-cells (*CCL5* ^+^, *GZMB* ^+^, *CD8A* ^+^), and platelets (SNCA^+^). **(B)** Dot Plot depicting expression profile of markers genes used for annotating different cell type clusters. The relative expression and percent of cells expressing specific markers are shown by shades of red color and the size of the dot respectively. **(C)** PBMC and SENSE single-cell method-based split UMAP showing the distribution of cell types. There are slightly elevated differences in T-cells subtypes in the SENSE group, while PBMC samples showed higher levels of myeloid cells. **(D)** Stacked bar plot showing the relative patient contribution in each individual cell type cluster. The samples from PBMC and SENSE methods are shown with shades of blue and red respectively. Each cluster depicted the varying levels of contribution from individual patients. The contribution of cells from each sample is shown using a pie graph with orange and purple colors representing SENSE and PBMC profiled samples respectively. **(E)** Heatmap displaying the top two gene markers expressed by each cell type. Columns represent individual cells, grouped by cell type, while rows display individual genes. Horizontal colored bars above the heatmap indicate the different cell types. Relative gene expression is shown in pseudo color, where blue represents low expression, and red represents high expression. Top markers generally correlate with well-established canonical markers for each cell type. **(F, G)** Comparative analysis proportions of cell types in the PBMC and SENSE methods. The proportion of Total **(F)** and CD45+ **(G)** cells per sample between PBMC and SENSE methods are shown. Each bar plot depicts the mean proportions and ± standard error of the mean. Each dot represents an individual sample. The significance of the difference in the mean in the groups was tested using paired Student’s t-test, with significant differences being indicated with * (*P*<0.05) and ** (*P*<0.01).

The high-quality cells obtained after filtration and normalization steps were clustered based on the gene expression profiles using Seurat (24). The initial 21 clusters obtained from the integrated scRNA-seq data of samples processed using PBMC and SENSE methods, were annotated to obtain 11 major cell types from various lineages using canonical marker genes: B-Cells (*MS4A1* ^+^, *CD79A* ^+^), Memory B-cells (*CD19^+^, IGLC2* ^+^), NK cells (*NKG7* ^+^, *KLRD1* ^+^, *CD3D* ^-^), Myeloid cells (*CD14^+^, MNDA^+^, FCGR3A^+^, FCN^+^*), CD4+ Naïve T-cells (*CD3D* ^+^, *CD4^+^, CCR7^+^, LEF1^+^*), CD4^+^ Memory T-cells (*CD3D* ^+^, *CD4^+^, TRAAD^+^, TNFRSF4^+^*), IFN T-cells (*CD3D* ^+^, *ISG15^+^, STAT1^+^, IFI6^+^*), CD8^+^ Effector T-cells (*GZMA* ^+^, *GZMB* ^+^, *CD8A* ^+^), CD8^+^ Naive T-cells (*CD8A* ^+^, *CCR7* ^+^, *LEF1* ^+^, *TCF7* ^+^), CD8^+^ Memory T-cells (*CCL5* ^+^, *GZMB* ^+^, *CD8A* ^+^), and platelets (*SNCA^+^*) (**Figures 3A, B**). Using the doublet detection algorithm of the DoubletFinder package (25), we identified two outlier clusters exhibiting doublet proportions greatly exceeding other clusters: Db 1 (95% doublets) and Db 2 (44.1% doublets). The remaining clusters demonstrated notably lower doublet percentages (averaging at 1.7 ± 0.8%). We reviewed the canonical markers expression in these doublet-enriched clusters to explore if they express markers of cell types from different lineages and correctly flagged doublets. Cluster Db1 highly expressed both pDC and T-cell related markers (*CD4, JCHAIN, MZB1, IRF8, CLEC4C*), whereas cluster Db2 highly expressed both plasma cell and T cell markers (*CD8A, JCHAIN, MZB1, CD38*). We also observed a small number of cells in both PBMC (n=120 cells) and SENSE (n=182 cells) methods that were enriched with mitochondrial genes (Mt Enriched) (**Figures 3A, B**). On average, T-cells were the largest cluster among all patients, followed by myeloid cells, and B-cells (**Figure 3A**). All the identified cell types (except platelets) were detected in samples processed using either SENSE or PBMC method (split UMAP in **Figure 3C**). Regardless of the processing method used, cells of the same type consistently clustered together, highlighting their high transcriptome similarity. The split UMAP visualization and bar plots depicted lower enrichment of cells from the myeloid lineage (PBMC: n=6,085, SENSE: n=1,903) and higher enrichment of T cells (PBMC: n=8,558, SENSE: n=15,373) in the SENSE method as compared to the PBMC method (**Figures 3C, D**). Most of the major clusters had contributions of cells from each patient, implying a similar single-cell landscape across all patients (**Figure 3D**). Some clusters, such as IFNγ responsive T-cells (IFN T-cells), showed a disproportionately high contribution from a single patient (patient 1) with both processing methods (**Figure 3D**), which may be a result of the clinical condition of that patient. Patient 1 is a recently diagnosed JIA patient as compared to other JIA patients in this study who have been undergoing treatment for quite some time (**Table 1**).

To further dissect the expression profiles of different cell types, we compared the gene expression profile of the target cluster with the other cells in the sample based on the non-parametric Wilcoxon Rank Sum test (average log-fold change (FC) ≥0.25, > 25% of cells expressing gene, and *P*< .01). This analysis allowed the identification of a gene signature for each cell type (**Figure 3E**). The SENSE method captured a higher number of T cells per sample as compared to the PBMCs method indicating its advantage in T-cell repertoire characterization (**Figure 3F**; **Supplementary Figure 2**). T cell subtype comparison depicted that CD8+ T cells (Naïve, Memory) are significantly elevated (*P*<.05) in the SENSE method profiled samples (**Figure 3F**; **Supplementary Figure 3**). On the other hand, the PBMC-based method depicted significant enrichment (*P*<.05) of the myeloid cells (**Figure 3F**). Additionally, PBMC method also depicted significant enrichment of platelets as compared to SENSE (*P*<.01) (**Figure 3F**). Similar observations were made while considering only CD45+ immune cells of samples, where the PBMC-based method illustrated significantly better capture for the memory B-Cells in addition to myeloid cells (**Figure 3G**). The disparity observed in myeloid cells and platelets may be attributed to the CD15+ granulocyte removal steps employed in the SENSE method, while the disparity in T cells might be due to density gradient step in the PBMC method.

### 2.3 Cell types exhibited similar transcriptome profiles for SENSE and PBMC methods

**Figure 4:**
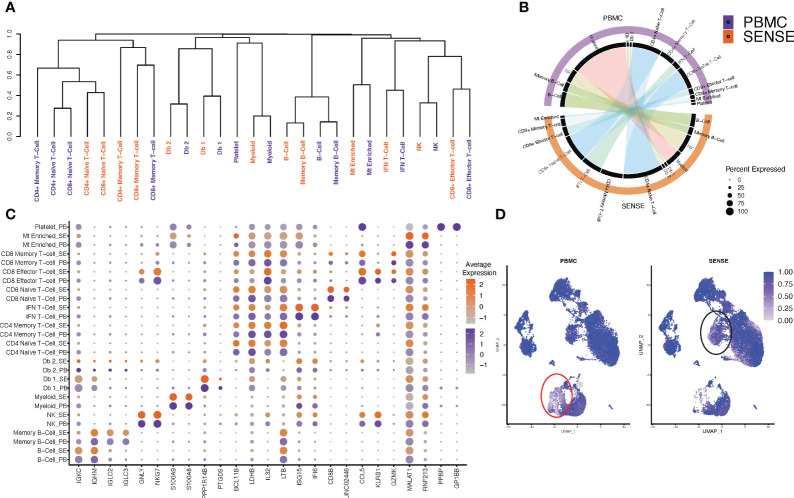
Comparison of single cell profiles of samples processed using SENSE and PBMC methods. The scRNA-seq data from blood samples processed using SENSE and PBMC methods were analyzed using a uniform bioinformatics workflow for comparative analysis. **(A)** Dendrogram showing the distances between cell types from each method based on the differentially expressed genes for each cell type computed independently. The differentially expressed genes were identified by comparing the target cell type with others based on an average log FC > 0.25 and Wilcoxon Rank Sum test *P*<.01 as well as genes expressed in > 25% of a given cell population. **(B)** A Circos plot showing the correlation between expression profiles of cell types profiled using SENSE and PBMC methods. The individual cell types between profiling methods depict significant similarities in the expression profiles. Some cell subtypes within the T cell compartment depicted lower correlations. **(C)** Comparative analysis of canonical cell type-specific markers between the two methods. Most of the cell type defining markers are concordantly expressed across corresponding cell types indicating strong similarity in the SCPs generated by SENSE and PBMC methods. The color scales on the right show the gene expression levels in samples processed using PBMC (purple) and SENSE (orange) methods. The size of the dot represents the percent of cells expressing specific markers. The Y-axis shows the cell types with SE indicating samples processed using the SENSE method and PB representing samples processed using the PBMC method. The X-axis shows the gene names. **(D)** Shannon’s entropy-based batch effect estimation. The UMAP plot shows Shannon’s entropy of different clusters calculated based on the distribution of SCP protocol labels (i.e., SENSE, PBMC) among the cell’s 100 nearest neighbors. The analysis was performed on normalized data without any batch effect correction. Low entropy values were observed in myeloid cell clusters (marked with red lasso) and an IFN T-cell cluster (marked with black lasso), indicating poor mixing and method-based batch effect.

To assess the sample processing method-induced technical variations in the overall expression profiles, we studied the clustering based on cell types split on SENSE and PBMC methods. Most of the matching cell types, irrespective of the processing methods, depicted similar clustering patterns except for subtle variations in the myeloid cells compartment (**Figure 4A**). The hierarchical clustering based on the cell types/subtypes markers genes identified based on the Wilcoxon Rank Sum test (average log-FC ≥0.25, >25% of cells expressing gene, and *P*< .01) depicted that naive and memory T-cells formed one distinct cluster, while doublets and myeloid cells formed another cluster. B-cells formed a separate cluster, and natural killer (NK) cells and effector T-cells clustered together in a different group. For most of the cellular compartments, the same cell types/subtypes depicted the highest correlation except the T cells compartment (**Figure 4A**). For example, the NK, CD8^+^ effector T-cells, and myeloid cells depicted the highest correlation between transcriptome profiles from SENSE and PBMCs method (**Figure 4A**). In the T cells compartment, some of the cell types depicted a weaker correlation between the matching cell types from the two methods indicating some variation. This finding aligns with a higher proportion of the T cells captured using the SENSE method as compared to PBMCs based method. To further validate the consistency of cell type labeling across methods, we assessed the similarity of the differentially expressed markers for each of the 11 cell types from the two methods. To achieve this, cells from each method were subsetted, and the top differentially expressed markers for each cell type with respect to all other cells from the same method were identified based on the Wilcoxon Rank Sum Test (average log-FC ≥0.25, >25% of cells expressing gene, and *P *< .01) and visualized using Circos plots generated using ClusterMap (26) R package. The cell types from the SENSE method depicted high transcriptome correlation with matching cell types from the PBMCs method, again indicating strong concordance among the methods (**Figure 4B**). Next, we assessed the similarity in canonical/top markers expression for various cell types based on processing protocol. The markers for each cell type depicted similar expressions irrespective of the processing method (split dot plot in **Figure 4C**). The consistency of key marker genes expression establishes the transcriptome similarity of cellular profiles from the SENSE and PBMCs methods. To further assess and quantify batch effects due to processing methods, we calculated Shannon’s entropy/cell to assess the degree of mixing of samples from the two methods (27). Low entropy values indicating poor mixing of a cells from different samples and methods were observed mainly in myeloid (0.526 ± 0.005) (**Figure 4D**, red lasso) and T cells (0.701 ± 0.004) (**Figure 4D**, black lasso). The rest of the cell types depicted high entropy (0.872 ± 0.001) indicating no batch effects (**Figure 4D**).

### 2.4 SENSE enables deep profiling of immune repertoire by capturing profiles of T cell subtypes

**Figure 5:**
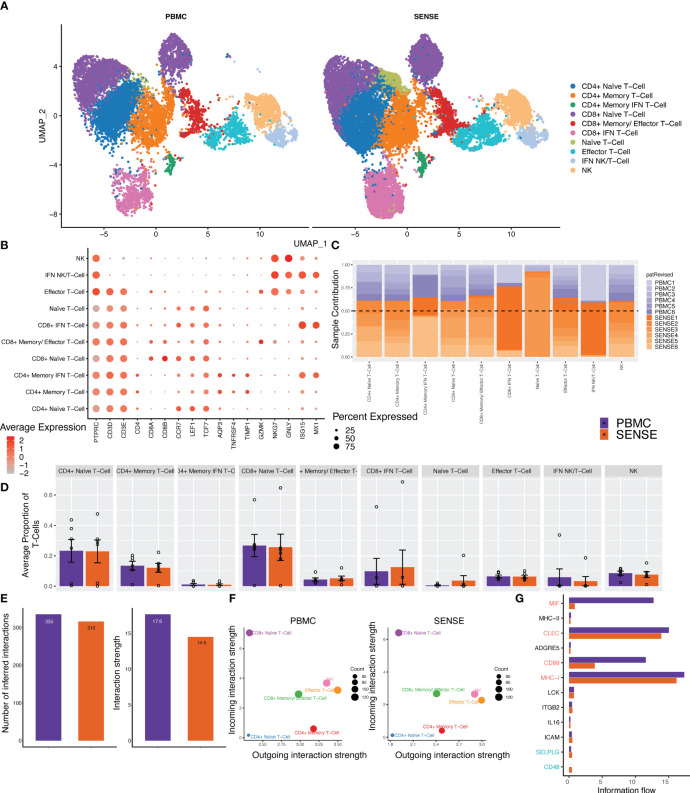
Focused analysis on the T cell clusters to estimate the impact of blood samples processing protocols. **(A)** A UMAP displaying the T-cell subclusters split based on single-cell processing protocols (i.e., SENSE, PBMC). Subclusters were manually labeled as T/NK cells (Naïve, Effector, IFN+, NK), CD4^+^ T-cells (Naïve, Memory, Memory IFN+), CD8^+^ T-cells (Naïve, Memory, IFN+) based on the expression of specific markers. The counts depicted slightly better capture for T cell sub-clusters in the SENSE method as compared to the PBMC method. **(B)** Dot plot demonstrating the expression profile of key markers for each T-cell subtype. The gradient of red color and size of dot represent the relative expression and percent cells expressing specific markers, respectively. **(C)** Stacked bar plot showing the relative patient contribution in each individual T- cell sub-cluster. The samples from PBMC and SENSE methods are shown with shades of blue and red respectively. Each cluster depicted the varying levels of contribution from individual patients. **(D)** Comparative analysis proportions of cell types in the PBMC and SENSE methods for T-cell subclusters. The proportion of total T-cells cells per sample between PBMC and SENSE methods for each sub-cluster is shown. **(E-G)** CellChat based analysis of cell-cell communication. **(E)** Total number of interactions and interaction strength of the inferred cell-cell communication networks for T-Cells from different methods, PBMC (purple) and SENSE (orange). **(F)** Scatter plot to compare the major sources and targets of interaction on the 2D space where the incoming and outgoing strength for each T cluster along the y-axis and x-axis, respectively. **(G)** Bar graph to compare the overall information flow of each signaling pathway between PBMC and SENSE methods.

T cells are highly diverse and play a critical role in eliciting immune responses against antigens. To further investigate the different T cell subtypes captured by SENSE and PBMC methods, we performed a focused analysis after subsetting out and reclustering the T and NK cell clusters. The analysis included 27,982 cells that were annotated into 10 distinct T and NK cell subtypes (**Figures 5A, B**) based on the expression of marker genes that include Naïve T-cells (*CD3D^+^, CCR7^+^, LEF1^+^*), Effector T-cells (*CD3D^+^, GNLY^+^, GZMK^+^*), CD4^+^ Naive T-cells (CD4^+^, CCR7^+^, LEF1^+^), CD4^+^ memory T-cells (*CD4^+^, TCF7^+^, TNFRSF4^+^*), CD4^+^ Memory IFN T-cells (*CD4^+^, TNFRSF4^+^*, *ISG15* ^+^, *MX1^+^*), CD8^+^ Naive T-cells (*CD8^+^*, *CCR7^+^, LEF1^+^*), CD8^+^ Memory/Effector T-cells (*CD8^+^, TCF7^+^, TNFRSF4^+,^GZMK^+^*), CD8^+^ IFN T-cells (*CD8^+^*, *ISG15^+^, MX1^+^*), IFN NK/T cells (*CD3D* ^-^, *GNLY^+^*, NKG*7^+^*, *ISG15^+^, MX1^+^*) and NK cells (*CD3D* ^-^, *GNLY^+^*, NKG*7^+^*). In both SENSE and PBMC methods various subtypes of CD8^+^ T-cells were the dominant T-cells (~41%), with the remaining cells consisting primarily of CD4^+^ T-cells (~37%) along with NK cells (~8%), other T cells (~8.5%) and NK/T (specific to patient 1, ~4.5%) (**Supplementary Figure 3**). The Naïve T cells formed 51% of the total T cells captured in the assays. Most of the T cell clusters had contributions from all the patients except IFN-stimulated clusters that are patient-specific (**Figure 5C**). Overall, the SENSE-based method captured a significantly (*P*=.007) higher number of T cells as compared to the PBMC method (**Supplementary Figure 2**), but the relative proportion of T cell subtypes is similar in both methods (**Figure 5D**; **Supplementary Figure 3**). Further to explore the functional landscape of T/NK we performed a comparative analysis of cellular communication based on the expression of ligands and receptors (28). Comparison of the overall number of interactions and their strengths revealed them to be similar between SENSE and PBMC methods (**Figure 5E**; **Supplementary Figure 4**). Further communication analysis depicted similar communication patterns among cell types, with CD8+ Naïve T-cells with the highest incoming interactions and Effector T-cells with the highest outgoing interactions (**Figure 5F**). Further, we explored the information flow of the signaling pathways based on the sum of communication probability among cell types of SENSE and PBMC methods. We observed that most of the pathways showed a similar information flow pattern, including CLEC, MHC-I, LCK, IL16, ICAM, and ITGB2 (**Figure 5G**). Some pathways including MIF, and CD99 depicted different signaling between cell types from SENSE and PBMC methods. These pathways typically involve myeloid, platelets, and dendritic cells (29–31). Therefore, the differential signaling observed in these pathways may be attributed to the differences in myeloid cells and platelets captured by the two methods. These results indicate a common signaling network operates between the cells processed using either of the two methods, indicating that the SENSE method yields similar results to the PBMC method and is suitable for analysis of the T cells landscape in whole blood samples.

### 2.5 Myeloid lineage cell types have lower enrichment but similar profiles between single-cell preparation methods

**Figure 6:**
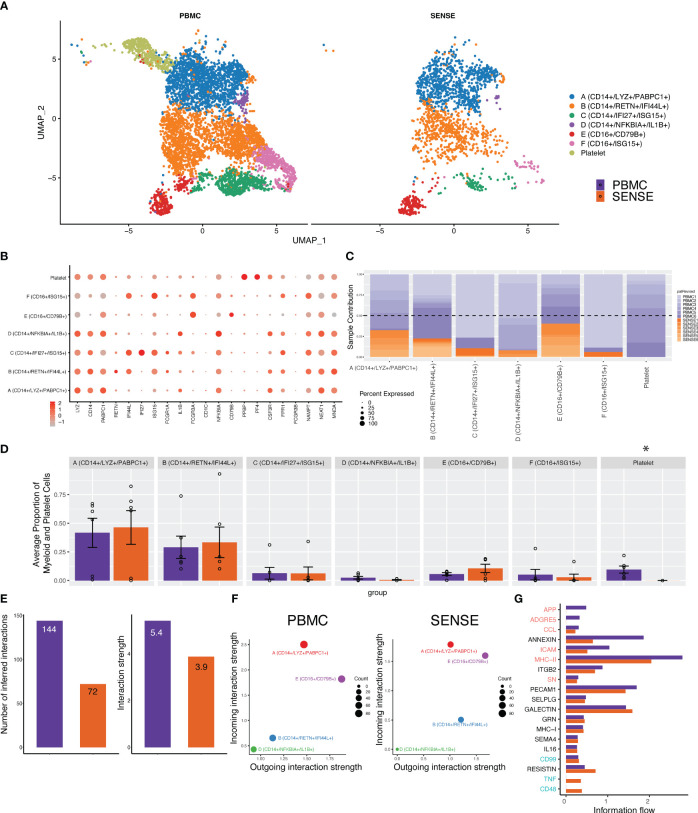
Focused analysis on the Myeloid cell clusters to estimate the impact of single-cell processing protocols. **(A)** A UMAP displaying the myeloid and platelet subclusters split based on single-cell processing protocols (i.e., SENSE, PBMC). Subclusters were manually labeled as Clusters A (*CD14+, LYZ+, PABPC1+*), B (*CD14+, RETN+, IFI44L+*), C (*CD14+, IFI27+, ISG15+*), D (*CD14+, NFKBIA+, IL1B+*), E (*CD16+, CD79B+*), F (*CD16+, ISG15+*), and Platelets (*PPBP+, PF4+*) based on the expression of top markers. The counts were lower for myeloid cell sub-clusters in the SENSE method as compared to the PBMC method. The platelets were present only in the PBMC samples. **(B)** Dot plot demonstrating the expression profile of common myeloid, neutrophils, and platelet markers. The gradient of red color and size of the dot represents the relative expression and percent cells expressing specific markers, respectively. **(C)** Stacked bar plot showing the relative patient contribution in each individual sub-cluster. The samples from PBMC and SENSE methods are shown with shades of blue and red respectively. Each cluster depicted the varying levels of contribution from individual patients. **(D)** Comparative analysis proportions of cell types in the PBMC and SENSE methods for myeloid and platelet subclusters. The proportion of total myeloid and platelet cells per sample between PBMC and SENSE methods for each sub-cluster is shown. **(E–G)**. CellChat based analysis of cell-cell communication for Myeloid clusters. **(E)** Total number of interactions and interaction strength of the inferred cell-cell communication networks for myeloid cells from different methods, PBMC (purple) and SENSE (orange). **(F)** Scatter plot to compare the major sources and targets of interaction on the 2D space where the incoming and outgoing strength for each T cluster along the y-axis and x-axis respectively, **(G)** Bar graph to compare overall information flow of each signaling pathway between PBMC and SENSE methods.

The myeloid cell compartment is the second largest compartment of cell types observed in both sample processing methods. Overall, the SENSE method captured a significantly lower number of myeloid cells (**Figure 3F**; **Supplementary Figure 2**) which might be due to the filtering out of CD15+ myeloid/granulocytes to enhance single cell capture efficacy and generate high-quality cells for capturing. The sub-clustering and annotation on the myeloid and platelet compartments comprising 7,599 cells yielded seven distinct cell types or subtypes. Among these, six were identified as myeloid subtypes and a single cluster as platelets. Both methods captured every cell type from the myeloid lineage as evident from the split UMAP plot (**Figure 6A**). These clusters look like classical and non-classical monocytes with the expression of *CD14* and *FCGR3A* genes along with the expression of other markers (**Figure 6B**). The analysis of patients’ contribution to different clusters depicted that some of the clusters like cluster A (*LYZ+, CD14+, PABPC1+*) had contributions from multiple patients whereas others like cluster F (*CD16+, ISG15+*) were patient specific (**Figure 6C**). Although the SENSE method captured fewer cells in this compartment, comparative analysis of relative proportions of myeloid cell subtypes depicts concordance of single cells captured using the two methods (**Figure 6D**). To better understand the communication patterns among different subtypes of the myeloid lineage, we conducted cell communication analysis. The analysis was performed only on the clusters that have cells from multiple patients. Our analysis revealed lower interactions and weaker strengths of interaction between the subtypes of the myeloid lineage using the SENSE method (**Figure 6E**). One possible explanation for these findings is that there were fewer overall cells present during the analysis in this compartment from the SENSE method (PBMC: n=5,401, SENSE: n=1,733). Comparison of cellular communications patterns among the myeloid cells depicted significant concordance with cluster A with characteristics like classical monocytes with most incoming interactions (**Figure 6F**). Further analysis of key pathways associated with the flow of information among cells also depicted multiple similar pathways including PECAM1, GRN, MHC-I, SELPLG, and Galectin (**Figure 6G**). In summary, although there is concordance in the myeloid cell sub types captured by the two methods, the lower capture of myeloid cells in the SENSE method is leading to some cellular communication differences that is one of its limitations.

### 2.6 SENSE method generated transcriptome profile similar to publicly available PBMC transcriptome profile

**Figure 7:**
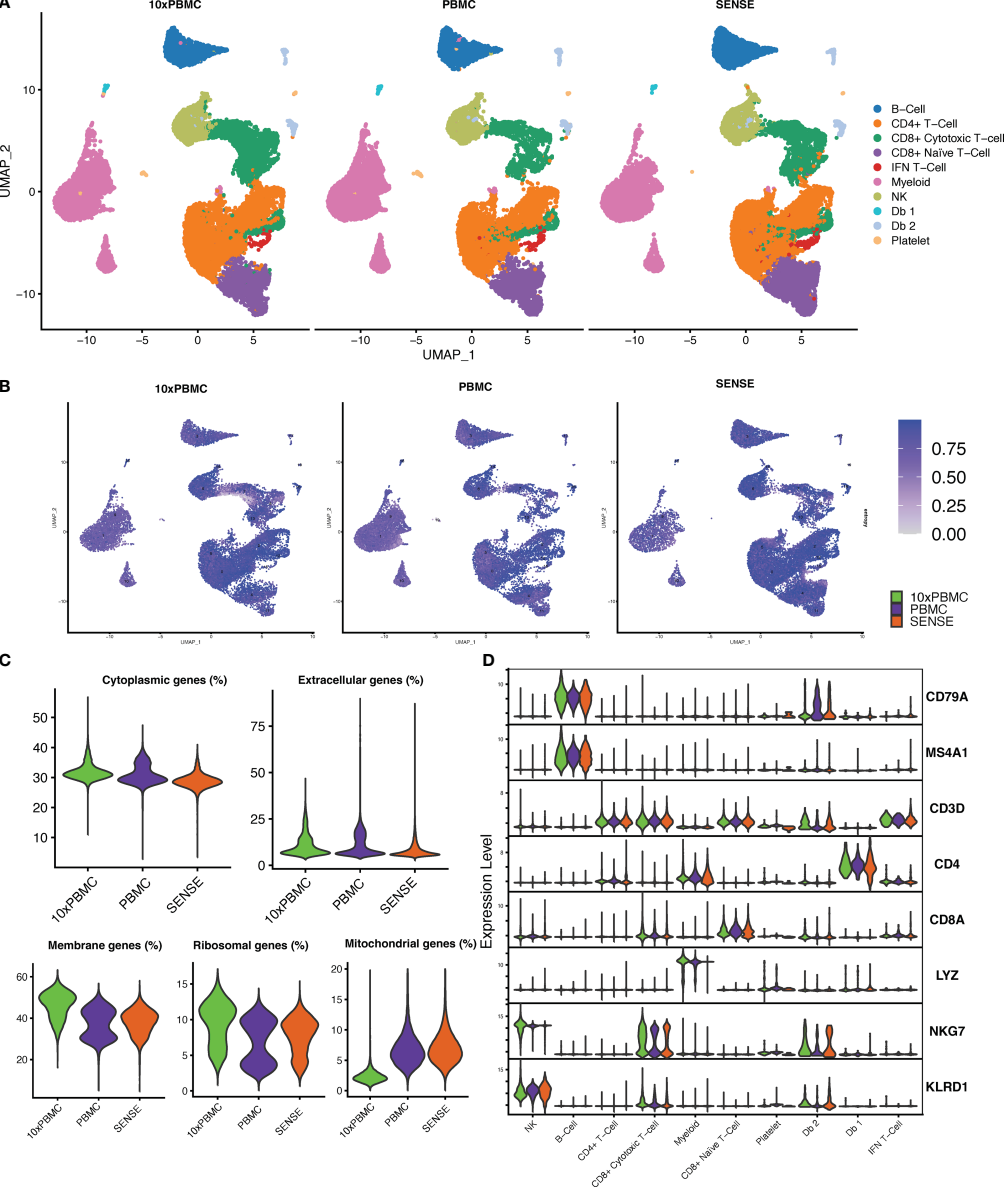
Comparing the transcriptomic profile of SENSE method samples with PBMC method samples from 10x Genomics legacy datasets. **(A)** Split UMAP of SENSE and PBMC methods data from our experiments and legacy data, 10x PBMC, from 10x genomics. Cluster labels are based on transferring the labels from SENSE, PBMC data. **(B)** Shannon’s entropy-based batch effect estimation. The UMAP plot shows Shannon’s entropy of different clusters calculated based on the distribution of SCP protocol labels (i.e., SENSE, PBMC, 10x PBMCs) among the cell’s 100 nearest neighbors. The analysis was performed on normalized data with batch effect correction using integration anchors. **(C)** Proportion of group-wise genes in cytoplasmic, membrane, extracellular, and ribosomal gene ontology categories, along with percent mitochondrial genes (green - 10x PBMC, purple-PBMC, orange - SENSE). **(D)** Violin Plots comparing the expression of various cell markers among our SENSE, PBMC data with 10x PBMC dataset.

To further evaluate the transcriptome profile of SENSE method WB generated data, we performed a comparative analysis with publicly available PBMC dataset. This PBMC dataset (32) was obtained from the 10x Genomics Inc. website and processed uniformly and integrated with our data using integration anchors-based batch correction. The comparative analysis of cellular profiles based on split UMAP depicted co-embedding of major cell types indicating similarity in transcriptome profiles (**Figure 7A**). In line with the publicly available 10x Genomics PBMC dataset (10x PBMC), the SENSE method also captured T cells as the most abundant cell types from the whole blood profiling. Shannon’s entropy was computed per cell to assess the degree of mixing of samples from three datasets (i.e., 10x PBMC, PBMC, SENSE). Most clusters from different datasets depicted high entropy indicating the mixing of cells from different datasets in respective clusters (**Figure 7B**). We observed low entropy in the myeloid cell clusters (i.e., poor mixing) which might be due to lower capture of myeloid cells using the SENSE method. Further comparative analysis of data quality by measuring proportions of cytoplasmic, extracellular, membrane, ribosomal, and mitochondrial genes depicted similar profiles indicating the similar quality of single-cell data (**Figure 7C**). The assessment of the similarity in canonical marker expression distribution from 10X PBMC dataset and our cells from SENSE method depicted similar expressions for most cell types (**Figure 7D**), with the primary exception being the previously noted myeloid cells. The consistency of key marker expression demonstrates that cell types can be identified reliably using the SENSE method, and comparative analysis can be performed among the samples profiled using different methods.

## 3 Discussion

Analysis of blood samples is the most direct and least invasive approach (33) to decipher disease mechanisms and identify biomarkers (34). SCP of blood samples is ideal for characterizing how the profiles and characteristics of different immune cells in the blood change in response to disease or therapy, however, the need for immediate sample processing to prepare and preserve viable single cells is a major deterrent towards implementing this on samples collected in a clinic or hospital setting. The traditional method for isolating PBMCs using the Ficoll-Paque density gradient method for SCP is cumbersome and its implementation is challenging due to the lack of time, equipment, and trained personnel in most clinics. This may also be partly responsible for the limited implementation of single-cell profiling in clinical trials. Direct cryopreservation of blood samples without pre-processing has been reported to result in cell death and RNA/DNA degradation, hampering molecular profiling (35). To address these limitations, we have developed the SENSE method for viably freezing WB collected in EDTA tubes without any need for centrifugation steps, special reagents, and trained personnel. The one-step addition of FBS/DMSO freezing solution assists in the cryopreservation of WB cells by preventing the formation of intracellular ice crystals, minimizing cell stress, and thereby maintaining cell integrity/preventing senescence. Granulocytes depletion post-thawing of WB samples enables the recovery of high-quality mononuclear cells as granulocytes are poorly cryopreserved in freezing media and release DNA and lysosomal enzymes promoting cellular damage/clumping (36).

Validation of one-step SENSE method for cryopreservation of WB will jump-start clinical implementation of SCP as well as advance single-cell research. To validate the SENSE method and demonstrate its suitability for cryopreserving high-quality single cells for SCP, we processed freshly collected blood samples with both the SENSE and the traditional density gradient isolation of PBMCs methods. The initial step following the procurement of blood samples, i.e., cryopreservation was much faster and easier with the SENSE method compared to the more time-consuming and complex density-gradient isolation of PBMCs. Although there were slight differences in the viability of cells after the thawing and washing steps, they were not significant and did not affect the quality of the single-cell profiles. We tested multiple quality metrics to evaluate the quality of cells prepared using the SENSE method in comparison to the PBMC isolation method. Quality metrics like median gene counts and unique molecular identifiers (UMIs), were found to be comparable between SENSE and PBMC methods. High mitochondrial content is indicative of poor-quality cells that are either undergoing apoptosis or have lyzed (37). The median representation of <10% mitochondrial genes in WB and PBMC samples confirms a similar proportion of high-quality single cells obtained with both methods. The SENSE method depicted a slight advantage in capturing the profile of the higher number of cells as compared to the PBMCs method from similar cell suspensions (concentration and viability). The high quality of cells was further confirmed by the lack of cellular damage artifacts with the SENSE method. On closer inspection of the percentage genes in the cytoplasmic ontology category, we see bimodal distribution in PBMC method, in contrast to a unimodal distribution in SENSE method (**Figure 2D**). Myeloid and platelet cells were found to have a higher percentage of cytoplasmic genes (~32% - 40%) as compared to the rest of the cell types (~20% - 30%) (**Supplementary Figure 5**). Myeloid and platelets cells were captured more in the PBMC method as compared to SENSE method, resulting in the bimodal distribution observed in **Figure 2D** with the former method. Processing times and cryopreservation can result in changes in cell cycle stages when performing SCP which will affect the transcriptome (38). We observed no significant differences in the cell cycle phases between samples processed using the two methods. Multiplets can be biologically misleading and arise when two or more cells are captured in single droplets during encapsulation steps (39). Doublets can occur due to poor quality of cells resulting in two cells clumping together, dying cells and/or broken cells resulting in misleading hybrid transcriptomes (25, 39). We did not observe any significant differences in the number of doublets present between the samples processed using the SENSE method and the PBMCs method. The SENSE method was shown to have slightly fewer doublets compared to the traditional PBMC method (doublet cells: PBMC method, n=916; SENSE method, n=539). Therefore, comparative analysis of the single-cell quality of cells obtained using the one-step cryopreservation SENSE method revealed striking similarities to the traditional multi-step cryopreservation PBMCs method, reinforcing its utility as a method of choice for ease of cryopreservation and single-cell profiling of clinical WB samples.

Cellular landscape revealed by clustering enables identification of cell types and their individual biological states and specific functional roles in disease development and progression (40). All major cell types were represented in UMAPs generated from scRNA-seq data of samples prepared with both methods. The marker genes were shown to have similar expressions for each cluster/cell type or subtype from both methods. Hierarchical clustering demonstrated that different immune cell types have unique transcriptomes that enable their classification into distinct clusters irrespective of sample processing method. The relative cellular abundance analysis revealed that while dominant cell clusters were similar in samples processed using either of the two methods, there were differences in the myeloid and T cells subtype clusters. Although the density gradient centrifugation method to get PBMCs should remove heavier granulocytes, there are instances of incomplete removal of granulocytes, especially in certain pathological conditions like sepsis (41) and autoimmune disorders (42) where there is increased amounts of low-density granulocytes (43). Also, delay in processing of blood can result in granulocyte activation; resultant degranulation gives rise to low density granulocytes that will not be separated out efficiently by density gradient methods (44). In SENSE method, the CD15^+^ granulocytes are selected and removed to obtain high quality CD15^-^ mononuclear cells. CD15+ cell depletion was combined with density gradient centrifugation to effectively purify PBMCs from sepsis patients with high percentage of low-density granulocytes (43). Though there was some resultant loss of additional cells other than granulocytes during the CD15^+^ cells depletion steps, the functional cellular properties were not compromised (43). The observed differences in myeloid clusters in this study might be attributed to the SENSE method’s removal of CD15^+^ cells to filter out sticky granulocytes that might have also filtered out aggregating monocytes and platelets. Importantly, the SENSE method was able to recapitulate the myeloid compartment associated with disease as we observed similar patient-wise differences of cell-type distribution in scRNA-seq data from both methods. In summary, even though SENSE captures fewer myeloid cells compared to the PBMC method, it is still capable of revealing differences in the proportions of myeloid cells within given samples.

On the other hand, we observed more efficient capture of T cells in SENSE method cryopreserved WB samples. Importantly, our focused analysis on the T cells compartment showed that the SENSE method captured a significantly higher number of T cells representing various types and subtypes, including Naïve, Effector, and Memory T-cells. The possible cause of lower T-cell enrichment with the PBMCs method might be due to some T-cells being lost during the Ficoll-Paque density gradient centrifugation step due to the difference in density of these cells (45). These results make our simple WB sample cryopreservation combined with CD15^+^ granulocyte removal method especially suitable for immune repertoire profiling using VDJ enrichment to explore the association of T cell clonality with disease or therapeutic outcomes analyses. Based on the single-cell quality metrics, the cryopreserved WB using the SENSE method yielded high-quality single cells similar to cryopreserved PBMCs isolated using the traditional density-gradient method. Furthermore, the SENSE method can be extended for more granular characterization of immune repertoire using single cell proteomics/multidimensional profiling.

Comparison analysis of our data with an external 10x Genomics PBMC dataset (32) revealed concordance between the three datasets as all cell types were consistently identified in all three datasets. The high quality of cells obtained with the SENSE method was further demonstrated by quality metrics like lower % membrane genes and higher % extracellular genes compared to the external PBMC dataset. This analysis further validates the robustness of the SENSE method to acquire high quality single-cells for single cell profiling.

## 4 Methods

### 4.1 Sample collection

Informed consent according to Emory University IRB protocol (IRB00079391 Determinants of Childhood Autoimmunity) was obtained from Juvenile Idiopathic Arthritis (JIA) (n=5) and pediatric Lupus (n=1) patients being treated in the rheumatology clinic in CHOA prior to sample collection. Blood samples were collected in lavender top EDTA tubes and transported to the lab from the clinic at room temperature. Samples were cryopreserved within 2h post-collection.

### 4.2 PBMCs isolation and whole blood cryopreservation

The freshly collected blood was split into two equal aliquots, with one aliquot processed for isolation of PBMCs while the other aliquot was frozen directly using a cryopreservation solution. PBMCs were isolated using the standard Ficoll-Paque density-gradient method according to manufacturer’s instructions (Cytiva). Briefly, blood diluted in phosphate buffer saline (PBS) (1:1) was gently layered onto Ficoll-Paque PLUS (Cytiva, 17144002) and spun at 500g for 30 minutes at 21°C. The top layer (plasma) was removed and discarded. The layer containing the mononuclear cells was then carefully removed and diluted with 3x volume of PBS, mixed well, and spun at 500g, for 10 minutes at 21°C. The pellet was resuspended in PBS and washed again by spinning for 10 minutes at 500g and 21°C. The PBMCs pellet was then resuspended in 1 ml recovery cell culture freezing media (Fisher Scientific, 12648010) at a concentration of <10X10^6^ cells/ml. The second set of blood samples (by the SENSE method) was viably preserved by mixing whole blood 1:1 with freezing solution made up of 80% heat-inactivated fetal bovine serum (hiFBS) and 20% dimethyl sulfoxide (DMSO). Samples were gradually frozen by placing in Mr. Frosty freezing container (Fisher Scientific, 5100-0001) and stored at -80 °C till further use.

### 4.3 Single cells preparation

Frozen PBMC samples were thawed and washed with wash buffer (PBS containing 1% BSA) to prepare viable single cell suspensions (14). Frozen whole blood samples were also thawed, and cells were pelleted (380g, RT, 6.5 minutes), the supernatant was gently removed so as not to disturb the pellet, which was then resuspended in EasySep buffer (STEMCELL technologies, 20144) and filtered through 100 µm filter mesh (Fisherbrand, 22363549). The EDTA concentration of EasySep buffer used for washing and diluting the cells was modified. The amount of EDTA in the EasySep buffer containing 1mM EDTA was increased to 4mM by adding an additional 3mM EDTA. EDTA is known to rapidly reverse the preferential binding of platelets to monocytes (46). Therefore, the presence of higher EDTA concentration in the buffer results in increased capture of high-quality mononuclear cells. Granulocytes were removed using a modified EasySep CD15 selection (EasySep™ Human CD15 Positive Selection Kit; STEMCELL technologies, 18651) protocol. Also, RBC depletion beads (STEMCELL Technologies, 18170) were added following the CD15 cocktail mix and RapidSpheres incubation steps to remove red blood cells. Following EasySep magnetic separation of CD15^+^ antibody-bound granulocytes, the CD15^-^ mononuclear cells supernatant was collected, cells pelleted and resuspended in PBS containing 1% BSA for generating viable cells for scRNA-seq libraries. A detailed stepwise protocol for the SENSE method is included as **Supplementary document 1**.

### 4.4 Single-cell assays and sequencing

ScRNA-seq libraries were prepared from viably thawed WB and PBMCs single-cell samples prepared in the previous section according to manufacturer’s (10x Genomics) instructions. CellPlex kit (10x Genomics, 1000261), which allows the pooling of samples prior to GEMs generation by labeling samples with unique cell multiplexing oligos (CMOs), was used to multiplex samples. The pooled samples were used to generate GEMs, followed by RT-PCR steps and cDNA amplification using Next GEM single cell 3’v3.1 kits (10x Genomics, 1000268). Following the cDNA amplification step, size selection beads were used to separate the CMO and gene expression (GEX) cDNAs that were then used to prepare the CMO and GEX libraries respectively. The final CMO and GEX libraries were then pooled and sequenced according to 10x Genomics sequencing parameters using Novaseq S4 PE100 (Illumina) kits for comprehensive transcriptome profiling.

### 4.5 Single-cell profiling analysis

The raw FASTQ files from each multiplex sample were aligned using 10x Genomics Cell Ranger (32) 6.1.2 to align against a reference human genome (GRCh38) for generating raw cell-gene count matrices. The count and CMO matrices from the samples were analyzed with R (v 4.2.2) using Seurat (47) (v 4.0.4) and other Bioconductor packages. Low-quality cells were filtered using Seurat to keep only cells with >200 unique genes, >600 UMI reads, and < 20% mitochondrial UMIs. Potential doublets were marked using the doubletFinder (25) algorithm that identifies doublets based on neighborhood search on principal component analysis (PCA). Assuming 3.5% of doublet formation from the 10x multiplexing experiment, we performed analysis with top 10 principal components with a neighborhood size of 0.1(pK) to predict doublets. The count matrices were normalized using the SCTransform algorithm, regressing out the per-cell UMI count, the number of unique features per cell, and the percent mitochondrial reads mapped to a cell. The normalized cell count was used for selecting the top 2,000 variable genes for principal component analysis (PCA) to identify the principal components capturing the most variance across the samples. Similar cells were clustered together via Louvain clustering on the top principal components using the Seurat package that was visualized Uniform Manifold Approximation and Projection (UMAP) to determine the overall relationship among the cells. The cell clusters were manually annotated based on canonical cell markers described in our previous studies. The cell markers for the different cell clusters were identified by comparing target cell types with others captured in the assay using the Wilcoxon Rank Sum test (adjusted *P*<.10, average log2FC > 0.25, and percent cell expression > 25%).

### 4.6 Entropy calculation and Gene-Ontology based cellular component enrichment

Shannon entropy was calculated per cell for assessing the batch effect due to method variation using 100 neighbors and 20 principal components using the CellMixS (27) R package. The gene signatures for cellular components (extracellular region, cytoplasm, membrane, ribosome) were sourced from Gene Ontology (48) database (GO:0005576, GO:0005737, GO:0016020, GO:0005840). PercentageFeatureSet function of Seurat was used to calculate the percentage of all UMIs that belong to the gene signature per cell.

### 4.7 Comparative analysis of cell types across single-cell processing methods

We performed a comparative analysis of cell type abundance as well as gene expression between SENSE and PBMCs methods. The cellular proportion per patient were calculated and compared between methods using paired t-tests. The cell types with a P value <.05 were considered significantly differently enriched between methods. To determine the correlation between cell types between methods we implemented the ClusterMap package in R designed to compare cellular profiles across multiple single-cell datasets (26). Initially differentially expressed genes (DEGs) for each cell type in a method-specific manner (i.e., SENSE, PBMC) were identified based on the fold change and Wilcoxon Rank Sum Test (average log-FC ≥0.25, >25% of cells expressing gene, and P <.01). DEGs were computed using Seurat’s “FindAllMarkers” function. This was followed by hierarchical clustering of DEGs using their presence or absence (binary expression) in different cell types to generate a cluster dendrogram. The relative distance of cell types on the cluster dendrogram can be quantified by the similarity of the cell types. The similarity of the cell types is measured based on their Jaccard index. To match a cell type profile with another cell type ClusterMap introduced a purity tree cut algorithm (26). The algorithm uses the origin of cell types, clustering pattern on the dendrogram, and similarity to match the cell types from different methods. This results in matching cell types as well as merging cell types in a group if cell types depict >90% similarity within a method. The results from the analysis are displayed as a Circos plot summarizing the similarity in cell types and subtypes similarity.

### 4.8 Cellular communication and interaction analysis

Cellular communication analysis was performed using the CellChat platform (28). Cells from each processing method were isolated, and ligand-receptor (L-R) analysis was performed on the SENSE and PBMC methods independently using the standard CellChat analysis. Differentially expressed signaling genes were identified using the Wilcoxon rank sum test (*P< 0.05*), which was followed by communication probability/strength calculation between any interacting cell types. The cell-cell communications were filtered out if they were present in a cell type/subtype with less than 10 cells. The number of interactions and their strengths were aggregated for each method. To compare the overall signaling structure between cells in SENSE and PBMC samples, interaction weights were used, which sum the information flow of all L-R interactions between two cell types of lymphoid and myeloid lineages. The sum of outgoing or incoming communication probability associated with each cell group was visualized on a scatter plot showing the dominant senders (sources) and receivers (targets) cell types. The size of the data points on the scatter plot corresponded to the number of inferred links, both outgoing and incoming, connected to each specific cell type. Information flow/interaction strength characterizes the likelihood of cell-cell interaction occurring through a given pathway. Cells with high expression of a known ligand will have high information flow scores with cells that have high expression of the matching receptor. The conserved or processing method-specific pathways were evaluated by comparing the sum of communication probability among cell-type pairs for each pathway.

### 4.9 Comparing the SENSE and PBMC data with external PBMC dataset

Single-cell gene expression dataset for frozen PBMC samples (10x PBMC) from 3 donors (Donor A, B, and C) were downloaded from 10x Genomics datasets (32). The filtered gene expression matrices were merged with the PBMC and SENSE samples. The count matrices were again normalized using the SCTransform algorithm, regressing out the per-cell UMI count, the number of unique features per cell, and the percent mitochondrial reads mapped to a cell. The top 2,000 variable genes were found, and further Louvain clustering was performed on the top principal components using the Seurat package that generated a UMAP to visualize the overall relationship among the cells. To correct for any batch effect the samples count matrices from 10x PBMC and PBMC, SENSE datasets were normalized and integrated using integration anchors-based batch correction approach of the Seurat package. The cell clusters were manually annotated by transferring cluster labels from PBMC, SENSE to 10x PBMC samples. Based on distribution of existing labels on new clustering, some clusters were merged like B-Cell (B-Cell and Memory B-Cell), CD4+ T-Cell (CD4+ Naïve and Memory T-Cell) and CD8^+^ Cytotoxic T-Cell (CD8^+^ Effector and Memory T-Cell).

## Data availability statement

The datasets presented in this study can be found in online repositories. The names of the repository/repositories and accession number(s) can be found below: https://www.ncbi.nlm.nih.gov/geo/, GSE226557(GEO).

## Ethics statement

The studies involving humans were approved by Emory University IRB protocol (IRB00079391 Determinants of Childhood Autoimmunity). The studies were conducted in accordance with the local legislation and institutional requirements. Written informed consent for participation in this study was provided by the participants’ legal guardians/next of kin.

## Author contributions

SS: Formal analysis, Methodology, Software, Writing – original draft, Writing – review & editing. BT: Formal analysis, Investigation, Methodology, Writing – original draft, Writing – review & editing. WP: Formal analysis, Software, Writing – original draft, Writing – review & editing. MB: Investigation, Writing – review & editing. LP: Investigation, Writing – review & editing. RP: Conceptualization, Writing – review & editing. SP: Investigation, Writing – review & editing. SB: Formal analysis, Writing – original draft, Writing – review & editing. DM: Conceptualization, Writing – review & editing. MB: Conceptualization, Formal analysis, Funding acquisition, Supervision, Writing – original draft, Writing – review & editing.
