# Supplementary material for: The Simple prEservatioN of Single cElls method for cryopreservation enables the generation of single-cell immune profiles from whole blood
Source: Front Immunol. 2023 Nov 28;14:1271800. doi: 10.3389/fimmu.2023.1271800 (PMC10713715; doi:10.3389/fimmu.2023.1271800)
Supplement: Supplementary file 1 [file DataSheet_1.docx]

***Supplementary Materials***

**Supplementary Document 1**

**SENSE method detailed protocol**

**I. Cryopreservation of whole blood samples**

**Materials**

# Phosphate-buffered saline (PBS) 1X (Corning, cat # 21-040-CV), heat-inactivated fetal bovine serum - hiFBS (Cytiva, SH309), dimethyl sulfoxide (DMSO) (Sigma, cat # D2650-100ml).

Cryovials (VWR, cat # 66021-974). Mr. Frosty (Thermo Scientific, cat # 5100-0001). The other materials are 15 ml tubes, tips, and pipettes for mixing and transferring.

**Before starting:**

Prepare the appropriate volume of the freezing solution - 80% hiFBS, 20% DMSO. Mix and keep on ice.

**Protocol**

1. Transfer blood collected in lavender top EDTA tubes to the lab at room temperature within 2-3 h of collection*^#^.
2. Label cryovials and keep them on ice.
3. Estimate the volume of blood collected, and transfer to a 15 ml tube.
4. Add equal volume (1:1) of freezing solution to whole blood. Mix gently by inverting 5-6 times.
5. Aliquot 1ml/cryovial** (the number of vials will be double the volume of blood collected, e.g., we obtain eight cryovials of viably frozen blood from 4 ml of collected blood).
6. Keep in Mr. Frosty at -80 ^o^C overnight.
7. For short-term storage keep at -80 ^o^C and for long-term storage transfer to a liquid nitrogen tank.

**Troubleshooting and tips**

* If plasma needs to be collected for additional analysis, the whole blood can be spun at 350g, 5 minutes, room temperature (RT), and the plasma fraction removed. Fresh blood cells may not pellet too well. Take out the upper plasma layer without disturbing the pellet. Add PBS equal to the volume of plasma removed and proceed to step II.

# Diluting blood with PBS can also be performed if the high lipid content of blood is of concern.

** The quality of cells can be measured pre/post freezing by checking the percent viability, using an automatic cell counter, or manually counting with a hematocytometer, as well as checking the cell morphology under the microscope.

**II. Isolation of mononuclear cells with depletion of granulocytes and RBCs from frozen whole blood samples for single-cell profiling**

**Materials**

# EasySep buffer (Stemcell technologies, cat # 20144), EasySep™ Human CD15 Positive Selection Kit( Stemcell Technologies, cat. No. 18651), EasySep magnet (Stemcell technologies, cat #18000), Red blood cell (RBC)-depletion beads (Stemcell Technologies Cat# 18170), EDTA (0.5M, pH 8.0, Invitrogen, cat # AM9260G), PBS 1X (Corning, cat # 21-040-CV), heat inactivated FBS - hiFBS (Cytiva, SH309), RPMI medium 1640 (1X) (Gibco, cat#11875093), DMSO (Sigma, cat # D2650-100ml).

1.5 ml DNA Lo-bind tubes (Eppendorf, Cat # 0030 108.51), 5ml DNA Lo-bind tube (Eppendorf, cat # 0030 108.310), 100 mm filter mesh (Fisherbrand, cat # 22363549), 40 µm Flowmi filter (Spectrum, cat # 974-24244), 50 ml tubes, 5 ml polystyrene round-bottom tubes (Falcon, cat # 352052). The other materials are tips and pipettes for mixing and transferring cells, and supernatants. Wide-bore 1 ml tips or precut 1 ml tips with a sterile blade to widen the opening, to use if needed.

**Before starting.**

**Bring reagents to 37 ^o^C**

- Complete RPMI (cRPMI) medium: 10% hiFBS in RPMI should be at 37 ^o^C.

**Keep at 4 ^o^C (ice-cold)**

- Washing and resuspension buffer (WRB): Either 0.5%FBS in PBS (no EDTA) or 1% BSA in PBS.
- Prewet 1.5ml tubes with 1ml 0.5%PBS/ 1% BSA in PBS and keep on ice.
- CD15 kit components.

**Bring to room temperature (RT)**

- Prepare modified EasySep buffer (mEasySep buffer*) with higher concentration EDTA buffer: EasySep Buffer (1mM EDTA) + 3mM EDTA = 4mM EDTA total, for example, add 60 µl 0.5 M EDTA/10 ml EasySep buffer.
- (opt) Prep Z.Green, AbMix

**Protocol**

1. Take 9 ml warm medium (cRPMI) into 50 ml tubes.
2. Thaw cryovial** in a 37 ^o^C water bath for 1-2 minutes (time for thawing is volume dependent), till a small ice crystal is left.
3. Add 1 ml of warm cRPMI to thawed cells and pour into the 50 ml tube containing warm cRPMI. Total volume is ~12 -13 ml.
4. Evaluate for the presence of floating aggregates (encapsulated “bubbles”) and try dissociating them by pipetting up and down using precut tips***.
5. Filter cells through 100 µm filter mesh into a new 50ml tube.
6. Rinse the old 50 ml tube with another 10 ml of warm cRPMI and pass through the filter, combining with filtrate from step 5 (swirling gently). Total volume is ~22 - 23 ml.
7. Spin for 6.5 min at 380g, RT.
8. Gently remove and discard supernatant with pipette****, flick the pellet to resuspend in remaining media, and gently re-suspend in 450 µl mEasySep Buffer (with 4 mM EDTA)*.
9. Filter through 40 µm mesh/filter and transfer to 5 ml Falcon tube. If needed mesh can be rinsed with an additional 50-100 µl of buffer. The final volume, with residual supernatant, can be up to 600 µl.
10. Add 45 µl of anti-CD15 beads (1/10 Vol) and incubate for 3 min at RT. Mix gently. Need not increase beads for volume up to 600 µl.
11. Add 45 µl of RapidSpheres (1/10 vol), mix gently, and incubate for 3 min.
12. Add 2 ml of EasySep Buffer. Add 60 µl RBC-depletion beads*****. Mix gently to evenly disperse them in the sample. There is no need for additional incubation, sample is immediately placed in the EasySep magnet for 4 min at RT.
13. Collect Neg1 sup into new 5ml tube by pouring into 5 ml lo-bind tubes without touching (2-3 sec).
14. Repeat steps 12 and 13, combining Neg1 with Neg2 fractions (total vol ~4.5 ml).
15. Spin for 10 min at 380 g, RT.
16. Flick the pellet and using 1ml pipette gently re-suspend the pellet with 0.8 ml of WRB.
17. Filter using a 40mm Flowmi filter into a 1.5 ml Eppendorf tube on ice.
18. Count the cells (10 µl) and (opt) take 50-100 µl for Staining.
19. Spin the cells in microcentrifuge for 10min at 400 g, 4 ^o^C.
20. Resuspend the cells in WRB to get an optimal concentration of 700-1200 cells/µl.

**Tips and troubleshooting**

* Prepare higher concentration EDTA containing EasySep buffer.

**If the cell number is low, then multiple aliquots of the same sample can be taken out and pooled for the single-cell assay.

***Steps 4,5: Sometimes, the samples may appear blob-like/contain bubbles at this step. Try to break it with gentle pipetting using precut 1 ml filter tips (cut the tip of 1 ml tips with a sterile blade). Using wide bore/precut tips is important.

****It is important to aspirate the supernatant and not decant it as the pellet can be soft.

*****RBC depletion: Check under the microscope for RBCs at step 8 and/or if the pellet at step 8 appears red, add RBC depletion beads to remove RBCs.

**Supplementary Figures**

**
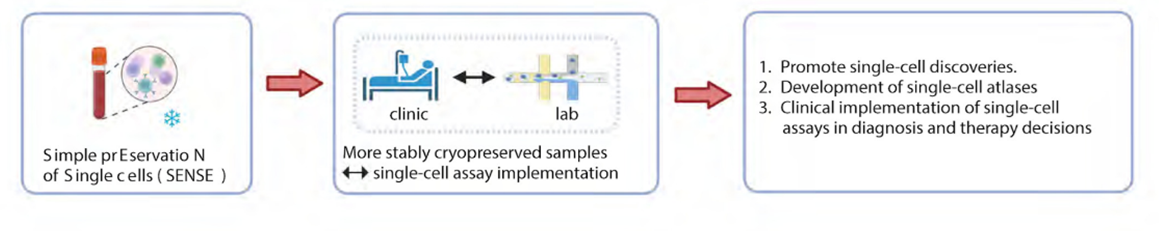
**

**Supplementary Fig. 1:** Advantages of the SENSE method in improving the clinic-to-bench sample transfer and bench-to-clinic single-cell assay implementation after optimization of simple one-step cryopreservation and single-cell profiling. The optimization of the novel SENSE method provides a valuable tool for the development of diagnostic/therapeutic strategies for a wide range of diseases. The figure was prepared using BioRender.

**
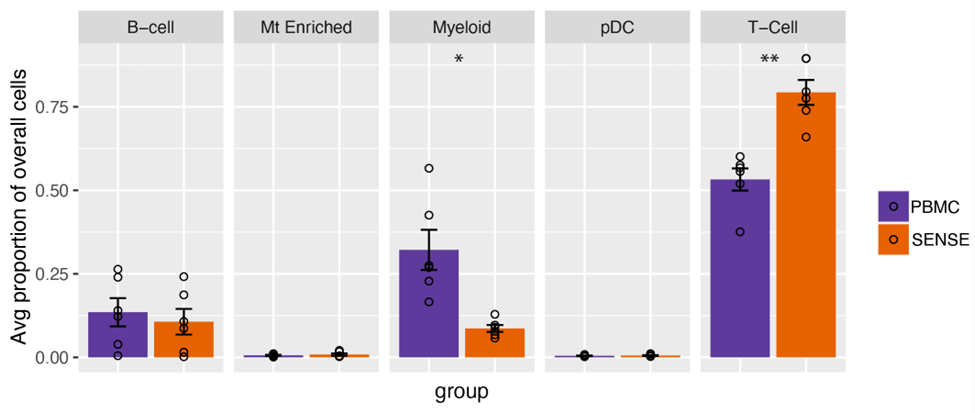
**

**Supplementary Fig. 2:** Bar plot of the average proportion of overall cells when comparing major immune cell types in cryopreserved PBMC samples (purple) and whole blood samples cryopreserved using the SENSE method (orange). *, *P*< .05 and **, *P*<.01.


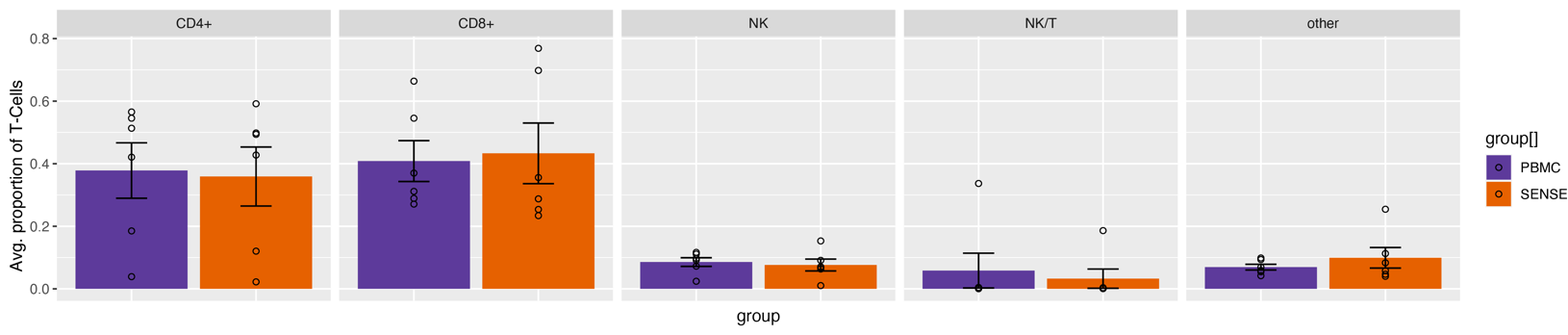


**Supplementary Fig. 3:** Bar plot of the average proportion of T-cells upon comparing major T-cell subtypes in cryopreserved PBMC samples (purple) and whole blood samples cryopreserved using the SENSE method (orange).

**Supplementary Fig. 4:** Bar plot of the average total number of interactions (Paired T-Test *P*=.7113) and interaction strength (Paired T-Test *P=*.6430) of the inferred cell-cell communication networks for subclusters of T-cells from different methods, PBMC (purple) and SENSE (orange). Each point on the bar plot corresponds to a single sample.

**Supplementary Fig. 5:** Percentage of cytoplasmic genes in different cell types in samples processed using PBMC (purple) and SENSE (orange) methods. X-axis represents cell types and Y-axis represents percent cytoplasmic genes.
